# Pax6 controls cerebral cortical cell number by regulating exit from the cell cycle and specifies cortical cell identity by a cell autonomous mechanism

**DOI:** 10.1016/j.ydbio.2006.08.035

**Published:** 2007-02-01

**Authors:** Jane C. Quinn, Michael Molinek, Ben S. Martynoga, Paulette A. Zaki, Andrea Faedo, Alessandro Bulfone, Robert F. Hevner, John D. West, David J. Price

**Affiliations:** a Genes and Development Group, Department of Biomedical Sciences, Centres for Integrative Physiology and Neuroscience Research, University of Edinburgh, Hugh Robson Building, George Square, Edinburgh, EH8 9XD, UK; b Stem Cell Research Institute, Dibit, H. S. Raffaele, Via Olgettina 58, 20132 Milan, Italy; c Department of Pathology, University of Washington, Seattle, Washington, WA 98104, USA; d Division of Reproductive and Developmental Sciences, Genes and Development Group, University of Edinburgh, Hugh Robson Building, George Square, Edinburgh, EH8 9XD, UK

**Keywords:** Chimera, Pax6, Proliferation, Telencephalon, Mouse, Tbr2, Mash1, Nkx2.1, Gsh2, Dlx2, Apical progenitor cell, Basal progenitor cell

## Abstract

Many cerebral cortical neurons and glia are produced by apical progenitors dividing at the ventricular surface of the embryonic dorsal telencephalon. Other neurons are produced by basal progenitor cells, which are derived from apical progenitors, dividing away from the ventricular surface. The transcription factor Pax6 is expressed in apical progenitors and is downregulated in basal progenitors, which upregulate the transcription factor Tbr2. Here we show that *Pax6*^*−/−*^ cells are under-represented in the cortex of *Pax6*^*+/+*^↔*Pax6*^*−/−*^ chimeras early in corticogenesis, indicating that Pax6 is required for the production of normal numbers of cortical cells. We provide evidence that this underproduction is attributable to an early depletion of the progenitor pool caused by greater than normal proportions of newly divided cells exiting the cell cycle. We show that most progenitor cells dividing away from the ventricular surface in *Pax6*^*−/−*^ embryos fail to express the transcription factor Tbr2 and that Pax6 is required cell autonomously for Tbr2 expression in the developing cortex of *Pax6*^*+/+*^↔*Pax6*^*−/−*^ chimeras. Transcription factors normally expressed ventrally in the telencephalic ganglionic eminences (Mash1, Dlx2 and Gsh2) are upregulated cell autonomously in mutant cells in the developing cortex of *Pax6*^*+/+*^↔*Pax6*^*−/−*^ chimeras; Nkx2.1, which is expressed only in the medial ganglionic eminence, is not. These data indicate that early functions of Pax6 in developing cortical cells are to repress expression of transcription factors normally found in the lateral ganglionic eminence, to prevent precocious differentiation and depletion of the progenitor pool, and to induce normal development of cortical basal progenitor cells.

## Introduction

Correct development requires regulation of the number of cells and the types of cell produced in each region. Regulating the numbers of postmitotic cells generated in the cortex requires control of two key aspects of proliferation: (i) the length of the cell cycle and (ii) the proportion of newly generated cells that re-enter the cell cycle as opposed to leaving it to differentiate. A number of cell cycle regulators and transcription factors, including Pax6, have been implicated in the control of these processes (Calegari and Huttner, 2003; Estivill-Torrus et al., 2002; Heins et al., 2002; Iacopetti et al., 1999; Roy et al., 2004). Two types of progenitor cell exist in the developing neocortex. Radial glia, also known as apical progenitor cells (APCs), divide at the ventricular surface either symmetrically, giving rise to two mitotic offspring, or asymmetrically to produce one mitotic and one postmitotic daughter [for review see Fishell and Kriegstein, 2003]. A second proliferative population, derived from the APCs, forms in the subventricular zone: the majority of these cells divide symmetrically to produce two postmitotic neurons, and they have been designated non-surface dividing cells or basal progenitor cells (BPCs) (Haubensak et al., 2004; Miyata et al., 2004; Noctor et al., 2004; Smart, 1976; Takahashi et al., 1995b). BPCs are thought to generate many of the neurons in the upper cortical layers (Altman and Bayer, 1990; Tarabykin et al., 2001). Pax6 is expressed in APCs and is downregulated in BPCs (Englund et al., 2005) and, in the present study, we addressed unanswered questions about the functions of Pax6 in the development of these cell types so as to generate a coherent model of the early functions of Pax6 in cortical development.

First, we examined how the overall production of *Pax6*^*−/−*^ cortical cells compared to that of *Pax6*^*+/+*^ cells early in corticogenesis. The fact that the dorsal telencephalon of *Pax6*^*−/−*^ embryos is smaller than that of wild types is not sufficient evidence for underproduction since it does not exclude the possibility that cells are more densely packed in the mutants, which is certainly the case in the later stages of corticogenesis (Caric et al., 1997; Kroll and O'Leary, 2005; Schmahl et al., 1993). We examined the production of *Pax6*^*−/−*^ cells in the cortex of *Pax6*^*+/+*^↔*Pax6*^*−/−*^ chimeras, allowing us to compare the numbers of cells with the two genotypes in the same animals and to test whether abnormalities persist even in the presence of wild-type cells, i.e., whether they likely reflect a cell autonomous requirement for Pax6. The results showed reduced production of mutant cells in our chimeras. We then investigated whether Pax6 is required to prevent excessive cell death, to regulate the length of the cortical progenitor cell cycle or to control the proportion of newly generated cells that re-enter the cell cycle as opposed to leaving it to differentiate. We found that the last of these parameters was altered in the *Pax6*^*−/−*^ cortex, indicating that Pax6 expression is required to maintain the size of the cortical progenitor pool. Next, we examined the BPCs in *Pax6*^*−/−*^ embryos. A recent study (Englund et al., 2005) showed that BPCs express the transcription factor Tbr2. The number of progenitors dividing away from the ventricular zone (or abventricularly) is increased in *Pax6*^*−/−*^ mutants (Estivill-Torrus et al., 2002; Haubst et al., 2004).We tested whether these cells resemble normal BPCs in expressing Tbr2 and found that the majority of abventricular mitoses in the *Pax6*^*−/−*^ mutant cortex did not express Tbr2. Since Pax6 is normally expressed in APCs and downregulated in BPCs, we determined whether Pax6 is required cell autonomously for Tbr2 expression using *Pax6*^*+/+*^↔*Pax6*^*−/−*^ chimeras.

The dorsal telencephalon of *Pax6*^*−/−*^ mutants becomes progressively ventralized throughout corticogenesis and this is due to a change in the fate of dorsal telencephalic progenitors (Kroll and O'Leary, 2005). What remains unclear is whether this fate change is a direct cell autonomous consequence of the loss of Pax6 in cortical progenitors or whether it results indirectly from a loss of Pax6 in interacting cells. We addressed this issue by examining the expression of ventral genes in mutant cells in the cortex of *Pax6*^*+/+*^↔*Pax6*^*−/−*^ chimeras.

## Methods

### Production of *Pax6*^*+/+*^↔*Pax6*^*−/−*^ chimeras

Chimeras used to estimate the numbers of mutant cells contributing to the cortex were produced as described in Quinn et al. (1996). In brief, eight-cell embryos were obtained from the parental cross *Pax6*^*Sey-Neu/+*^, *Gpi1*^*b/b*^ female × *Pax6*^*+/Sey*^, *Gpi1*^*b/b*^, *Tg/Tg* male, where Tg denotes the presence of the reiterated β-globin transgene TgN(Hbb-b1)83Clo (Keighren and West, 1993; Lo et al., 1987). Embryos of the following four genotypes were obtained from this parental cross: *Pax6*^*+/+*^, *Pax6*^*Sey-Neu/+*^, *Pax6*^*+/Sey*^ and *Pax6*^*Sey-Neu/Sey*^, all of which were *Gpi1*^*b/b*^ and contained a single copy of the β-globin transgene (Tg+). Donor embryos for aggregation were obtained from (BALB/c x A/J) F_2_ intercrosses, producing embryos that were *Pax6*^*+/+*^, *Gpi1*^*a/a*^ and negative for the β-globin transgene (Tg−). Embryos were collected from superovulated females at 2.5 days post coitum and aggregated according to West and Flockhart (1994). Aggregated embryos were cultured overnight, transferred to recipient pseudopregnant F1 females (*Pax6*^*+/+*^, *Gpi1*^*c/c*^, Tg−).

To increase the efficiency of return of *Pax6*^*+/+*^↔*Pax6*^*−/−*^ chimeras for subsequent studies of the identities of *Pax6*^*−/−*^ cells, we derived *Pax6*^*−/−*^ mutant embryonic stem (ES) cells from mice on an inbred 129/Sv background that were hemizygous for the β-globin transgene TgN(Hbb-b1)83Clo (Keighren and West, 1993; Lo et al., 1987) (designated 129SeyD). 129SeyD1 ES cells (*Pax6*^*−/−*^, *Gpi1*^*a/a*^, Tg+) were injected into (C57Bl/6 x CBA/Ca) F^2^ intercross blastocysts (*Pax6*^*+/+*^, *GpiI*^*b/b*^, Tg−), which were transferred to the uterus of a pseudopregnant recipient and allowed to develop to the appropriate embryonic stage.

Fetuses were dissected into cold PBS and staged according to forelimb development (Palmer and Burgoyne, 1991; Wanek et al., 1989). Tail and forelimb samples were taken for analysis of glucose phosphate isomerase (GPI1) isotype contribution to give a proportion of global chimerism for each embryo (West and Flockhart, 1994). A mean percentage GPI1 was determined to give the proportion of cells in the chimera derived from the *Pax6*^*Sey-Neu/+*^ × *Pax6*^*+/Sey*^ 8-cell embryo or 129SeyD1 ES cells. The genotype of each chimera was determined by PCR and restriction digest analysis of genomic DNA as described previously (Quinn et al., 1996). The use of two predicted null mutant Pax6 alleles, *Pax6*^*Sey*^ and *Pax6*^*Sey-Neu*^ (Hill et al., 1991), allowed distinction between aggregation chimeras containing *Pax6*^*Sey-Neu/Sey*^ compound heterozygous (described here as *Pax6*^*−/−*^) cells and those containing *Pax6*^*Sey-Neu/+*^ or *Pax6*^*+/Sey*^ heterozygous (described as *Pax6*^*+/-*^) cells. Chimeric embryos obtained by ES cell injection were genotyped for the presence of the *Pax6*^*Sey*^ allele alone. Histological visualization of cells derived from the *Pax6*^*Sey-Neu/+*^ × *Pax6*^*+/Sey*^ embryos or from 129SeyD1 ES cells was achieved by DNA–DNA in situ hybridization using a digoxigenin-labeled probe to the reiterated β-globin transgene (Keighren and West, 1993; Quinn et al., 1996). Detection of in situ signal was achieved either with peroxidase-labeled antibody visualized with diaminobenzidine (DAB) (Keighren and West, 1993) or by reaction with an anti-digoxigenin rhodamine antibody (Roche). We observed no phenotypic differences between *Pax6*^*+/+*^↔*Pax6*^*−/−*^ chimeras obtained by aggregation or ES cell injection.

### Quantitative analysis of contribution of *Pax6*^*−/−*^ cells to *Pax6*^*+/+*^↔*Pax6*^*−/−*^ chimeras

Percentages of Tg+ cells in various regions of the forebrain and other tissues of E12.5 chimeras were measured in a minimum of three non-consecutive sections, with 300–700 cells counted per area per section, depending on tissue. As in previous studies (Quinn et al., 1996), observed percentages for each tissue (O) were corrected to allow for failure to identify Tg+ signals in all Tg+ cells due to sectioning artefact. To generate tissue-specific correction factors (c), percentages of Tg+ nuclei in *Pax6*^*+/+*^, Tg+ embryos (i.e., non-chimeric embryos in which all cells should be Tg+) were counted at E12.5. Corrected observed percentages of Tg+ cell contribution (Oc) for each chimeric tissue were then divided by the percentage of cells expected in that tissue (E) if the percentage was to equal the global percentage chimerism estimated by GPI1B analysis (Oc/E). Values were compared by Student's *t*-test.

### Production of *Pax6*^*−/−*^ mice

All non-chimeric mouse embryos designated *Pax6*^*−/−*^ were derived from *Pax6*^*Sey*^ heterozygote crosses maintained on an inbred Swiss background. Wild-type siblings were used as controls. The day of the vaginal plug following mating was designated E0.5. Pregnant females were killed by cervical dislocation. Fetuses were dissected from the uterus at the required gestational age before fixing and processing to either wax or plastic sections. Wax sections were cut at 10 μm and plastic sections at 5 μm.

### Quantitative analysis of cell densities and cortical depth

In all cases, images were captured using Leica NTS confocal microscopy. Areas for cell density analysis were defined using Image-Tool™. Cells were counted on a minimum of 5 non-consecutive sections at E12.5. Cell counts/densities were compared using Sigmastat™. Cortical depths were measured in the center of the neocortex using Image Tool and data compared using Sigmastat™.

### Studying cell cycle with iododeoxyuridine (IdU) and bromodeoxyuridine (BrdU)

On embryonic day E10.5 or E12.5, IdU was injected i.p. into pregnant dams followed by BrdU injection (both at 70 μg/g body weight) 1.5 h later (Fig. 2A). Dams were killed at 2.0 h after the first injection; embryos were fixed, sectioned and processed to reveal IdU/BrdU using mouse monoclonal anti-BrdU (Becton Dickson Ltd., UK), which recognized both IdU and BrdU, in conjunction with rat monoclonal anti-BrdU (Abcam Ltd., UK) which recognized BrdU alone. Directly conjugated AlexaFluor® secondary antibodies were anti-mouse AlexaFluor® 488 and anti-rat AlexaFluor® 568. Images were captured using Leica NTS confocal microscope, viewed using LCS Lite (Leica) and imported into Adobe Photoshop for counting. Proportions of IdU/BrdU-labeled cells in the ventricular zone of telencephalon were counted in a minimum of three (E10.5) or five (E12.5) non-adjacent sections from each embryo. Cell cycle lengths were calculated using the following paradigm (Martynoga et al., 2005): cells in the initial IdU-labeled cohort that leave S-phase during the interval between IdU and BrdU (*T*_*i*_ = 1.5 h), designated the leaving fraction (*L*_cells_), will be labeled with IdU but not BrdU. The proportion of cells labeled with BrdU is designated *S*_cells_. The length of S-phase (Ts) can be calculated using the formula:

$$\frac{\mathrm{Ts}}{1.5}=\frac{S_{\text{cells}}}{L_{\text{cells}}}$$

and the length of the cell cycle (Tc) estimated from the formula:

$$\frac{\mathrm{Tc}}{Ts}=\frac{P_{\text{cells}}}{S_{\text{cells}}}$$

where *P*_cells_ is the total number of proliferating cells in the ventricular zone (VZ) (Martynoga et al., 2005). Since previous studies have shown that a prolonged pulse of BrdU will label virtually all VZ cells at E12.5, in both wild-type and *Pax6*^*−/−*^ embryos, *P*_cells_ was estimated by counting all VZ cells (Estivill-Torrus et al., 2002).

### Cumulative BrdU analyses

BrdU was given (70 μg/g body weight, i.p.) to E12.5 pregnant dams either once or every 2 h over a 12-h period. Dams were killed 0.5 and 12.5 h after the first injection; embryos were fixed, sectioned and processed to reveal BrdU as described previously (Gillies et al., 1990). The relative intensity of BrdU label in the nucleus of each BrdU-labeled cell in three 200-μm-wide strips through the cortex, in three non-adjacent sections from each embryo, was measured using a Leica digital camera and QWin (Leica) software.

### Immunocytochemistry on cortical cells

Cells from E12.5 neocortex of *Pax6*^*+/+*^ and *Pax6*^*−/−*^ embryos were dissociated using papain as per manufacturer's instructions (Papain Dissociation System, Worthington Biochemicals, UK) and stained for β-tubulin isotype III (mouse monoclonal IgG2b, 1:100, Sigma, UK). Visualization was achieved using directly conjugated AlexaFluor® 488 (goat anti-mouse IgG, 1:200) or AlexaFluor® 546 (goat anti-mouse IgG, 1:200) (Molecular Probes, Inc.). For cell counts, 800–1500 viable cells per culture were assessed in six randomly selected microscope fields.

### Flow cytometric analysis of cortical cells

Cortices were dissociated as above and fixed in ice cold 70% ethanol. Cortical tissue was collected from E12.5 and E14.5 embryos from 4 separate litters and a minimum of 3 individuals of each genotype pooled at dissection. Dissociated cells were stained for β-tubulin isotype III (1:800); primary antibody binding was revealed using directly conjugated AlexaFluor® 488 (1:800) as above. Cells were then stained with propidium iodide (PI) to allow discrimination of single cells and analysis of DNA content. Staining reactions were carried out in duplicate. Cells were analyzed on a Beckman-Coulter XL flow cytometer with Expo32 software (Beckman-Coulter, Inc.). 8000–20,000 cells were analyzed per sample.

### Cell death analysis

Cells from E12.5 neocortex of *Pax6*^*+/+*^ and *Pax6*^*−/−*^ fetuses were dissociated and cultured for 24 h as described previously (Estivill-Torrus et al., 2002). Cultures contained cells of either genotype alone or both genotypes mixed, with one set of cells stained using PKH26 fluorescent cell linker (Sigma) according to the manufacturer's instructions. Cells were fixed with 4% paraformaldehyde and visualized for localization of activated caspase-3 using rabbit polyclonal anti-caspase-3 antibody (Chemicon International; 1:100). Secondary amplification was with anti-rabbit biotin-conjugated antibody (DAKO; 1:200) and visualization was with streptavidin-conjugated AlexaFluor® 488 (Molecular Probes; 1:200). Cells were counterstained with bisbenzimide. Apoptotic cells were identified either by immunoreactivity for activated caspase-3 activity (Lesuisse and Martin, 2002; Srinivasan et al., 1998) and/or nuclear chromatin condensation, as described previously (Kerr et al., 1972). Cell counts were done in six randomly selected microscope fields per culture.

### Microarray hybridization

Total RNA from E14.5 neocortex was isolated using a method based on guanidinium lysis and phenol–chloroform extraction (ToTALLY RNA, Ambion). Labeling of total RNA was performed using the dendrimer technology (3DNA Submicro Expression Array Detection Kit, Genisphere). The cDNA was then hybridized to a cDNA chip representing 1026 different genes of the TESS subtractive cDNA library (Faedo et al., 2004) which had been generated by subtracting genes expressed in adult telencephalon from those expressed in E14.5 cortex (Porteus et al., 1992). Differential gene expression was assessed by scanning the hybridized arrays as described previously (Faedo et al., 2004). Changes in Tbr2 gene expression were confirmed at a protein level by Western blotting for Tbr2 protein expression using standard protocols. Equal loading of lanes was confirmed using β-actin immunostaining.

### Immunohistochemistry

Embryos were fixed overnight in 4% paraformaldehyde/PBS and processed to wax. All embryos were sectioned in the coronal plane. Slides were microwaved in 10 mM sodium citrate to achieve maximal antigen retrieval before addition of primary antibody. Antibodies used were mouse monoclonal anti-β-tubulin isotype III (Sigma, UK), mouse anti-phosphohistone 3 (Abcam Ltd., UK), rabbit polyclonal Tbr2 (Englund et al., 2005), mouse anti-Mash1 (BD Biosciences), rabbit anti-Dlx2 antibody (Abcam Ltd., UK), anti-Gsh2 (Toresson et al., 2000), anti-Nkx2.1 (Biopat) and directly conjugated AlexaFluor® secondary antibodies (Molecular Probes). Nuclei were stained with TOPRO3 (Molecular Probes). Where necessary, signal amplification was achieved using either Dako ABC or Dako Envision Kit prior to staining with DAB.

## Results

### Cortical thickness is reduced at E12.5 in the *Pax6*^*−/−*^ mutant embryo

A reduction in cortical thickness is a reported feature of the homozygous *Pax6*^*−/−*^ telencephalic phenotype during mid-late corticogenesis (Caric et al., 1997; Fukuda et al., 2000; Haubst et al., 2004; Schmahl et al., 1993; Warren et al., 1999). To determine whether reduction had occurred by E12.5, we measured thickness in the center of the neocortex of *Pax6*^*−/−*^ embryos and their wild-type siblings. Neocortical thickness was significantly reduced in *Pax6*^*−/−*^ embryos (*Pax6*^*+/+*^cortex: mean cortical depth 135 μm ± 6.2, SEM; *Pax6*^*−/−*^ cortex: mean cortical depth 114 μm ± 7.1, SEM; *n* = 9 in both cases; Student's *t*-test, *p* < 0.05).

### *Pax6*^*−/−*^ cells are under-represented in the dorsal telencephalon of E12.5 *Pax6*^*−/−*^↔*Pax6*^*+/+*^ chimeric mice

To directly compare *Pax6*^*−/−*^ and *Pax6*^*+/+*^ cell production in vivo, we created *Pax6*^*−/−*^↔*Pax6*^*+/+*^ chimeras. There are two advantage of this approach. First, it allows a direct comparison of the productivity of mutant and wild-type cells in the same embryo. Second, abnormalities in the production of *Pax6*^*−/−*^ cells in chimeras are attributable to a cell autonomous requirement for Pax6 in progenitors (Pratt et al., 2002; Quinn et al., 1996; Talamillo et al., 2003). In *Pax6*^*−/−*^ embryos, an absence of Pax6 could affect progenitors indirectly, as a consequence of a primary effect on other cells (a cell non-autonomous effect), but this is less likely in chimeras where mutant cells can interact with wild-type cells.

**Fig. 1:**
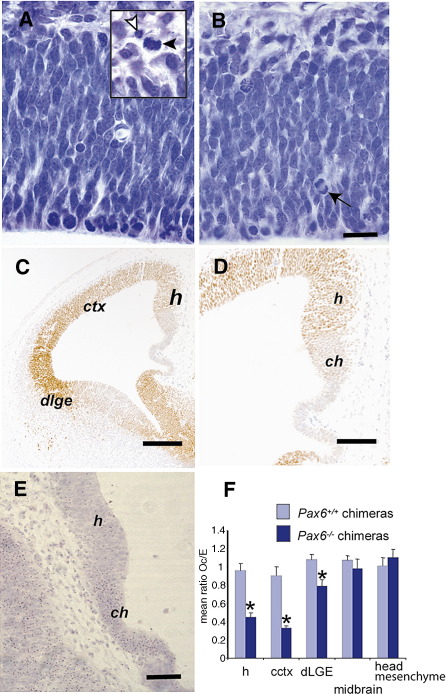
*Pax6*^*−/−*^ cells are under-represented in the telencephalon of E12.5 *Pax6*^*−/−*^↔*Pax6*^*+/+*^ chimeras. Plastic embedded sections of (A) *Pax6*^*+/+*^ and (B) *Pax6*^*−/−*^ E12.5 cortex. Boxed area in panel A is an enlargement of preplate of wild-type neocortex showing a cell undergoing mitosis (closed arrowhead) next to a cell undergoing apoptosis (open arrowhead). Mitotic figures were seen at the ventricular surface and at subventricular locations in both *Pax6*^*+/+*^ and *Pax6*^*−/−*^ cortex (e.g., arrow in panel B). (C) Pax6 protein localization in the E12.5 *Pax6*^*+/+*^ telencephalon. Pax6 is expressed in a ^high^lateral to ^low^medial gradient in the ventricular zone of the developing neocortex (ctx), hippocampus (h) and dorsal lateral ganglionic eminence (dLGE). (D) High power image of medial telencephalon showing boundary of Pax6 expression between hippocampus and cortical hem (ch). (E) *Pax6*^*−/−*^↔*Pax6*^*+/+*^ chimera (with a global contribution of 53% *Pax6*^*−/−*^, Tg+ cells determined by GPI1 analysis) showing under-representation of *Pax6*^*−/−*^, Tg+ cells (brown spots in nucleus) in the Pax6-expressing hippocampus but not in the cortical hem. (F) Composition of telencephalic regions of E12.5 *Pax6*^*+/+*^↔*Pax6*^*+/+*^ and *Pax6*^*−/−*^↔*Pax6*^*+/+*^ chimeras. Mean ratios ± SEM of corrected percentages of Tg+ cells in each tissue to global GPI1B percentage in the embryo (Oc/E) are shown for both *Pax6*^*+/+*^↔*Pax6*^*+/+*^ (light bars) and *Pax6*^*−/−*^↔*Pax6*^*+/+*^ (dark bars) chimeras. At E12.5, there is a significant reduction in the number of *Pax6*^*−/−*^ cells contributing to hippocampus (Student's *t*-test, *p* < 0.001), neocortex (*p* < 0.03) and dLGE (*p* < 0.02) in *Pax6*^*−/−*^↔*Pax6*^*+/+*^ (dark bars) chimeras. Significant differences are marked with an asterisk (*). Scale bars: (A, B) = 20 μm, (C) = 200 μm, (D) = 100 μm, (E) = 50 μm.

We generated chimeras in which a β-globin transgene (designated Tg) labels one of the two sets of cells used to make the chimeras (*Pax6*^*−/−*^ cells in *Pax6*^*−/−*^↔*Pax6*^*+/+*^ chimeras or one set of *Pax6*^*+/+*^ cells in *Pax6*^*+/+*^↔*Pax6*^*+/+*^ control chimeras). Telencephalic regions known to express Pax6 in wild-type embryos were examined (Figs. 1C, D). Observation of sections of E12.5 *Pax6*^*−/−*^↔ *Pax6*^*+/+*^ chimeras indicated lower densities of *Pax6*^*−/−*^ cells in Pax6-expressing regions than in Pax6 non-expressing regions. Fig. 1E shows an example of this. The density of *Pax6*^*−/−*^ cells (marked with dark dots) in the Pax6-expressing hippocampus was lower than the density of *Pax6*^*−/−*^ cells in the cortical hem, in which the majority of cells are Pax6 non-expressing (Fig. 1D).

The proportions of Tg+ cells in various tissues were then quantified in these chimeras. Regions analyzed were Pax6-expressing hippocampus, neocortex, dorsolateral ganglionic eminence (dLGE) and Pax6 non-expressing midbrain and head mesenchyme. If Pax6 status was neutral in terms of the generation of cell number in these regions, we would expect the percentages of Tg+ cells found in each structure in all chimeras to be similar to the overall percentage of Tg+ cells throughout the embryo; this expected percentage (designated *E*) was estimated for each chimera as described in Methods. The percentages of Tg+ cells observed in the structures studied, corrected for sectioning artefact (Oc), were obtained and the ratio Oc/E was calculated for each region analyzed in each chimera (see Methods). As inclusion of the Tg marker is developmentally neutral (West et al., 1996), we would expect this ratio to be close to 1.0 in all structures in *Pax6*^*+/+*^;Tg+↔*Pax6*^*+/+*^ chimeras and in structures that do not normally express Pax6 in *Pax6*^*−/−*^;Tg+↔*Pax6*^*+/+*^ chimeras. Mean Oc/E ratios (± SEMs, *n* = 4 in all cases) are represented in Fig. 1F and agree with this expectation. Mean Oc/E ratios were significantly lower in hippocampus, neocortex and dLGE in *Pax6*^*−/−*^;Tg+↔*Pax6*^*+/+*^ chimeras than in *Pax6*^*+/+*^;Tg+↔*Pax6*^*+/+*^ chimeras (Fig. 1F).

### Cell death is not increased early in corticogenesis in the *Pax6*^*−/−*^ neocortex

One possible explanation for the reduced size of the *Pax6*^*−/−*^ mutant cortex and the under-representation of *Pax6*^*−/−*^ cells in *Pax6*^*−/−*^↔*Pax6*^*+/+*^ chimeras was increased cell death. Although previous studies found no increase in the proportions of apoptotic cells in the cortex of *Pax6*^*−/−*^ mutant embryos (Estivill-Torrus et al., 2002; Fukuda et al., 2000; Lotto et al., 2001; Warren et al., 1999), these studies were at later stages of development. We found very few pyknotic cells in the E10.5–E12.5 cortex of either (i) *Pax6*^*−/−*^ embryos (Figs. 1A, B) or (ii) *Pax6*^*−/−*^↔*Pax6*^*+/+*^ embryos (e.g., none are seen in Fig. 1E). Since the proportion of dying cells at a given time depends on clearance of cells from the tissue, which in this case is unknown and may vary between genotypes, we carried out a further comparison of viability *in vitro*, where clearance cannot occur. There were no significant differences in the proportions of apoptotic *Pax6*^*−/−*^ and *Pax6*^*+/+*^ E12.5 neocortical cells after 24 h when the two types of cell were cultured either separately or mixed in a 1:1 ratio. In the mixed cultures, percentages of apoptotic cells identified (i) by nuclear morphology were 9.16% (± 0.55, SEM, *n* = 4) among *Pax6*^*+/+*^ cells and 11.14% (± 0.93 SEM, *n* = 4) among *Pax6*^*−/−*^ cells and (ii) by activated caspase-3 were 7.75% (± 0.77 SEM, *n* = 4) among *Pax6*^*+/+*^ cells and 7.53% (± 0.87 SEM, *n* = 4) among *Pax6*^*−/−*^ cells. Proportions of apoptotic cells were also examined in cells acutely dissociated from E12.5 neocortex of *Pax6*^*−/−*^ and *Pax6*^*+/+*^ embryos by staining with PI and using flow cytometry. The proportion of cells in a sub-G1 peak (cells with a hypodiploid DNA content) can be used to indicate the proportion of cells undergoing apoptosis (Ormerod, 2000). Sub-G1 fractions were not significantly different between *Pax6*^*−/−*^ mutant and *Pax6*^*+/+*^ neocortex (*Pax6*^*+/+*^ neocortex: 0.45% ± 0.13 SEM, *n* = 4; *Pax6*^*−/−*^ neocortex: 1.43% ± 0.59 SEM, *n* = 3, Student's *t*-test *p* = 0.119).

### Cell cycle length is not altered in E10.5 and E12.5 *Pax6*^*−/−*^ telencephalon

**Fig. 2:**
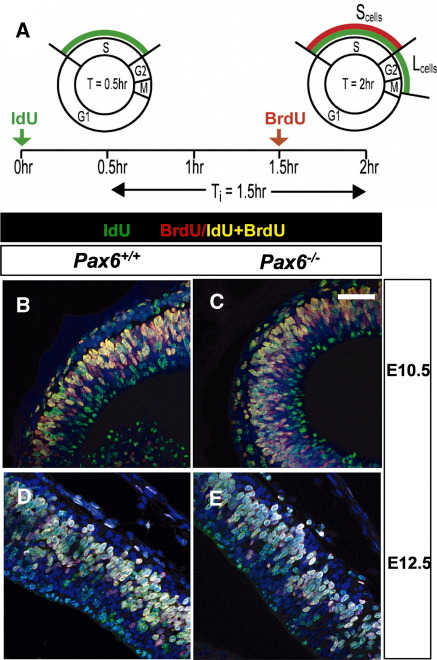
Analysis of cell cycle characteristics using IdU/BrdU co-labeling. (A) Injection at T0 h with IdU and T1.5 h with BrdU followed by sacrifice at T2.0 h allows identification of three cell types: those within S-phase at T0–1.5 but within G2/M at T1.5–2.0 (*L*_cells_: IdU-labeled only—green in panels B–E); cells entering S-phase at T1.5–2.0 (BrdU-labeled only—red in panels B–E); cells within S-phase at both T0 and T1.5 (IdU/BrdU co-labeled—yellow in panels B–E). By counting the numbers of cells in each of these three populations, cell cycle characteristics can be determined as described in Methods. Cell cycle characteristics were examined at E10.5 (B, C) and E12.5 (D, E) in wild-type (B, D) and *Pax6*^*−/−*^ (C, E) telencephalon. Scale bar = 50 μm.

Under-representation of Pax6 mutant cells in the cortex of *Pax6*^*−/−*^↔*Pax6*^*+/+*^ chimeras in the absence of increased cell death among mutant cells increased the likelihood that the explanation for the reduced production of *Pax6*^*−/−*^ cells results from a cell autonomous alteration in some aspect of their cell cycle. Paradoxically, previous studies indicated that the cell cycle of *Pax6*^*−/−*^ telencephalic progenitors is abnormally rapid at E12.5 (Estivill-Torrus et al., 2002; Warren et al., 1999), which might increase cell production. We re-investigated cell cycle length in the *Pax6*^*−/−*^ telencephalon using a method illustrated in Fig. 2A(Martynoga et al., 2005). Cell cycle lengths for wild-type and mutant telencephalon are shown in Table 1. Consistent with previous studies (Takahashi et al., 1995a), cell cycle lengths increased between E10.5 and E12.5. At E10.5 similar values for cell cycle length were observed in wild-type and mutant dorsal telencephalon (Table 1, Figs. 2B, C). At E12.5, no significant difference was observed in cell cycle length between mutant and wild-type neocortex or hippocampus (Table 1; Figs. 2D, E). At E12.5, cell cycle lengths in the hippocampus were longer than in the neocortex of both mutant and wild-type animals (Table 1). No significant differences were observed between wild-type and mutant embryos in the ratio between the length of S-phase and the entire cell cycle length (Ts/Tc) in either hippocampus or neocortex (Table 1). The reasons why this method gave a different outcome to that used previously (Estivill-Torrus et al., 2002) are presented in Discussion. In any case, neither this nor the previous estimates provide evidence that a lengthening of the cell cycle is the cause of an underproduction of *Pax6*^*−/−*^ cells.

### Loss of Pax6 reduces S-phase re-entry and increases the proportions of differentiating neurons

**Fig. 3:**
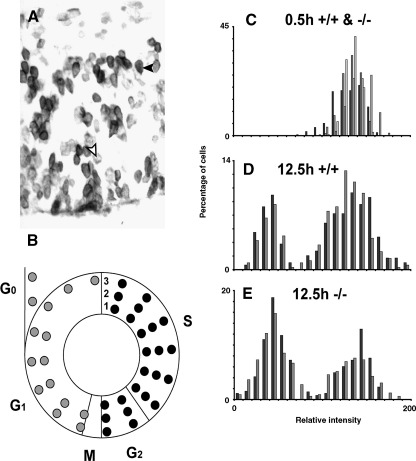
Assessment of proportions of cells exiting the cell cycle using cumulative BrdU labeling. (A) Section through the neocortex of an E12.5 *Pax6*^*+/+*^ embryo showing BrdU-labeled cells after a 12.5-h pulse of BrdU-labeled cells are either heavily (closed arrowhead) or lightly labeled (open arrowhead). (B) Diagrammatic representation of the cell cycle indicating how proportions of heavily and lightly labeled cells would vary for progressively longer pulses of BrdU. After a pulse shorter than the length of time between S-phase and M-phase (example 1), a single population of heavily labeled cells would be identified (black circles). After a longer pulse (example 2), a second population of labeled cells with a lower mean intensity of label would emerge as cells progress through M-phase (grey symbols). As the pulse lengthens further (example 3) cells in the second, lightly labeled population would either re-enter S-phase and replenish their BrdU load and become heavily labeled or would exit the cell cycle, enter G0 and remain lightly labeled. (C–E) Graphs illustrate frequency distributions for intensity of BrdU label in cells in histological sections after pulses of BrdU lasting (C) 0.5 h and (D, E) 12.5 h in wild-type (C, D) and mutant (C, E) neocortex. Each set of differently shaded bars corresponds to an individual animal. (C) At 0.5 h in both mutant and wild type all cells are heavily labeled with BrdU. (D, E) After a 12.5-h pulse with BrdU, heavily and lightly labeled cell populations can be identified but more lightly labeled cells are present in the mutant (E) than in wild type (D).

The most likely explanation remaining for a reduction in the production of *Pax6*^*−/−*^ telencephalic cells is premature withdrawal of newborn cells from the cell cycle, causing an increase in the proportion of differentiated cells with concurrent depletion in the size of the progenitor pool. Evidence that proportions of dividing cells exiting the cell cycle were higher in *Pax6*^*−/−*^ neocortex was obtained by measuring the proportions of lightly and heavily BrdU-labeled cells (Fig. 3A) in the ventricular zone after administering BrdU to E12.5 embryos for either 30 min or continuously over 12.5 h. Immunocytochemical detection of the amount of BrdU contained within cells is a well-established method for monitoring progression through the cell cycle in flow cytometry experiments (Ormerod, 2000). Similarly, previous studies in tissue sections have shown that a pulse of BrdU given to an animal results in cells with different relative levels of BrdU labeling. Cells in S-phase at the time of the pulse remain relatively heavily labeled unless they undergo subsequent divisions, in which case their levels of BrdU are diluted and their labeling becomes relatively lighter (del Rio and Soriano, 1989; Gillies and Price, 1993; Price et al., 1997). Here, the method was used as follows. After a short pulse of BrdU, cells in S-phase and cells that had just left S-phase would be detected by heavy labeling (Fig. 3B, example 1). After longer pulses, a more lightly labeled population of cells would emerge as cells carrying BrdU divide in M-phase and distribute their BrdU load between their daughters (Fig. 3B, example 2). The proportion of cells in the lightly labeled population would increase with increasing length of BrdU pulse until a maximum is reached as some cells re-enter S-phase and incorporate more BrdU (Fig. 3B, example 3). We predicted that if more cells become postmitotic in *Pax6*^*−/−*^ mutants than in wild-type embryos, then proportions of lightly labeled cells would be higher in mutants after a continuous 12.5-h pulse of BrdU, which is long enough to allow proliferating cells to re-enter S-phase. Our data are consistent with this prediction (Figs. 3C–E). After a short pulse (0.5 h), normal distributions of labeling intensity were obtained from both genotypes (Fig. 3C). After longer pulses (12.5 h), both *Pax6*^*+/+*^ and *Pax6*^*−/−*^ embryos showed bimodal distributions, but in the *Pax6*^*−/−*^ embryos the proportions of lightly labeled cells were higher than in *Pax6*^*+/+*^ embryos (Figs. 3D, E).

**Fig. 4:**
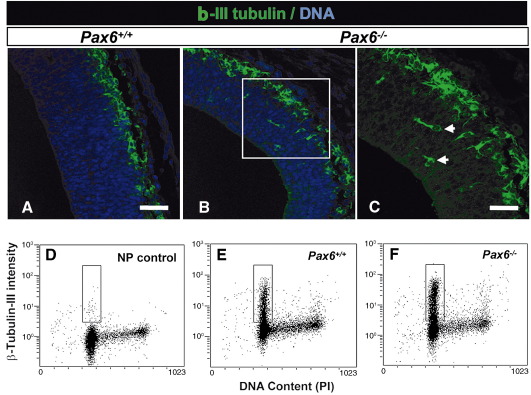
Precocious neurogenesis in the E12.5 *Pax6*^*−/−*^ telencephalon. β-III-tubulin expression in (A) wild-type and (B, C) *Pax6*^*−/−*^ neocortex. (A) In wild-type telencephalon at E12.5, β-III-tubulin-expressing cells are present in the preplate. (B) In *Pax6*^*−/−*^ telencephalon, β-III-tubulin-expressing neurons can be observed both in the preplate and abnormally located within the ventricular zone (white arrowheads in panel C). (C) Enlargement of area outlined in panel B. (D–F) Flow cytometric analysis of β-III-tubulin expression in dissociated (E) *Pax6*^*+/+*^ and (F) *Pax6*^*−/−*^ E12.5 neocortical cells. (D) No primary (NP) control reaction: gating was established (boxed region in panels D–F) by PI staining for cells containing a diploid DNA content (cells in G1/G0). Plots shown are from a single representative experiment for each genotype. The proportion of cells expressing β-III-tubulin are within the boxed region. (F) *Pax6*^*−/−*^ neocortex shows an increase in the number of β-III-tubulin-expressing neurons. Scale bars = (A, B) 80 μm; (C) = 40 μm.

If more cells are exiting the cell cycle from E10.5–E12.5, we predicted a corresponding increase in the proportion of differentiated neurons in *Pax6*^*−/−*^ telencephalon by E12.5. To confirm this, we examined the expression of the early neuronal marker β-III-tubulin in wild-type and *Pax6*^*−/−*^ neocortex (Figs. 4A–C). We observed β-III-tubulin-expressing cells located within the VZ of the mutant neocortex (Figs. 4B–C) and it appeared that the proportions of β-III-tubulin-expressing cells were increased, especially given the overall thinning of the cortical wall (Figs. 4A, B). To quantify this, we dissociated E12.5 cortical cells and counted the proportions of β-III-tubulin-labeled and unlabeled cells. Manual counts showed a significant increase (Student's *t*-test; *p* < 0.025) in the proportions of β-III-tubulin-expressing cells (mean = 38.7% ± 3.4% SEM, *n* = 3) compared to *Pax6*^*+/+*^ neocortex (mean = 23.2% ± 2.5% SEM, *n* = 3). This was confirmed using flow cytometry (*Pax6*^*−/−*^ neocortex: 29.43 ± 1.68% SEM, *n* = 3; *Pax6*^*+/+*^ neocortex; 19.15 ± 1.21% SEM, *n* = 5; Student's *t*-test *p* < 0.005). Figs. 3D–F show representative flow cytometry plots demonstrating this effect. It also reveals that the expression of this marker is restricted mainly to cells with a diploid DNA content, suggesting that β-III-tubulin expression within the cell cycle is not significantly altered by loss of Pax6 expression.

DNA staining using PI in flow cytometry experiments identified a significant decrease in the proportions of cells in S-phase in *Pax6*^*−/−*^ neocortex (*Pax6*^*+/+*^ neocortex; mean: 30.62%, ± 1.09 SEM, *n* = 5; *Pax6*^*−/−*^ neocortex: 24.73%, ± 1.31 SEM, *n* = 3; Student's *t*-test *p* = 0.011), consistent with a relative depletion of the *Pax6*^*−/−*^ mutant progenitor pool. Thus, three lines of evidence using different techniques all pointed to the conclusion that a larger proportion of cells exit the cell cycle and differentiate in the absence of Pax6.

### The transcription factor Tbr2 is downregulated in the *Pax6*^*−/−*^ neocortex

**Fig. 5:**
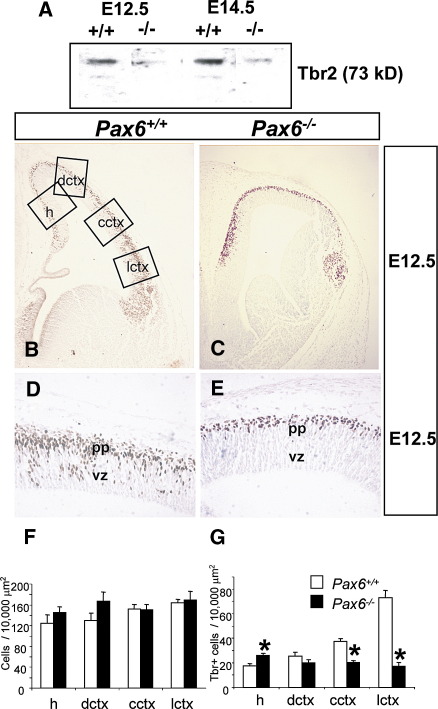
The transcription factor Tbr2 is downregulated in the *Pax6*^*−/−*^ telencephalon. (A) Western blot for Tbr2 protein from E12.5 and E14.5 cortex of *Pax6*^*+/+*^ and *Pax6*^*−/−*^ embryos. A significant reduction in Tbr2 protein is observed in the homozygous mutant lateral cortex at both E12.5 and E14.5. (B–E) Tbr2 localization in *Pax6*^*+/+*^ (B, D) and *Pax6*^*−/−*^ (C, E) telencephalon at E12.5. In *Pax6*^*+/+*^ telencephalon, Tbr2-expressing cells are located in the preplate (pp) and within the ventricular and subventricular zone (vz). In the *Pax6*^*−/−*^ telencephalon, almost all Tbr2-expressing cells are present in the preplate. (F) Quantification of telencephalic cell density and (G) Tbr2-expressing cell density in hippocampus (h), dorsal cortex (dcxt), central neocortex (cctx) and lateral neocortex (lctx) (boxed areas in panel B) at E12.5 in *Pax6*^*+/+*^ (open bars) and *Pax6*^*−/−*^ mutant (filled bars) telencephalon. (F) No significant difference is observed in cell density between mutant and wild-type animals in any telencephalic region examined. (G) A significant increase is observed in the density of Tbr2-expressing cells present in the *Pax6*^*−/−*^ hippocampus and a significant decrease in the densities observed in the *Pax6*^*−/−*^ central neocortex and lateral neocortex compared to wild types. Significant differences (Student's *t*-test, *p* < 0.05) are marked with an asterisk (*).

Microarray analysis was performed to screen for candidate genes up- or downregulated in *Pax6*^*−/−*^ neocortex (Faedo et al., 2004). The transcript that was most reduced at E14.5 was that of the T-domain containing transcription factor Tbr2 (Bulfone et al., 1999; Kimura et al., 1999) (downregulated by a factor of 2.52). Downregulation of Tbr2 protein was confirmed earlier, at E12.5, as well as at E14.5 in total protein isolated from *Pax6*^*+/+*^ and *Pax6*^*−/−*^ neocortex (Fig. 5A).

Tbr2 is expressed in both postmitotic neurons and BPCs in the dorsal telencephalon (Englund et al., 2005). Localization of Tbr2 protein in *Pax6*^*−/−*^ mutant and wild-type neocortex was compared (Figs. 5B–G). In wild-type E12.5 dorsal telencephalon, Tbr2-expressing cells were found mainly in the preplate with some in the proliferative zone (Fig. 5B,D) in a ^high^lateral to ^low^medial density gradient (Fig. 5B). This distribution of Tbr2-labeled cells closely mirrors expression of Pax6 in the ventricular zone (Fig. 1A; see also Englund et al., 2005). In the E12.5 *Pax6*^*−/−*^ cortex, almost all Tbr2-expressing cells were in the preplate (Figs. 5C, E) and very few were in the underlying proliferative zone. The ^high^lateral to ^low^medial gradient of Tbr2-expressing cells was lost (Fig. 5C); this was confirmed by quantitation of Tbr2-expressing cell density (Fig. 5G). The gradient was flattened: there was a small but significant increase in the number of Tbr2-expressing cells in the mutant hippocampus while lateral cortical regions showed large reductions in the number of Tbr2-expressing cells (Fig. 5G). These changes were not explained by overall differences in total cell densities between wild-type and mutant telencephalon in any area examined (Fig. 5F). Thus, expression of Tbr2 is strongly downregulated in the proliferative cells of mutants.

### Examining the nature of Tbr2-expressing cells in *Pax6*^*−/−*^ neocortex

**Fig. 6:**
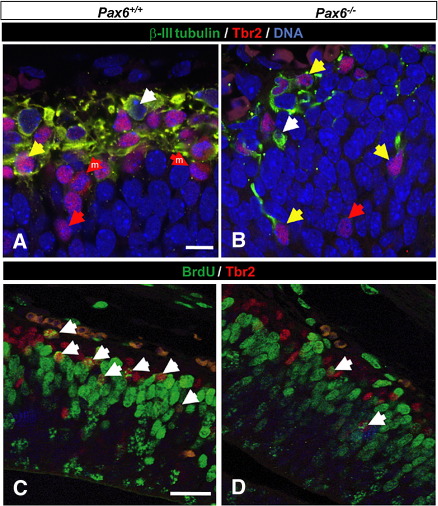
Tbr2-expressing postmitotic neurons are present in the *Pax6*^*−/−*^ telencephalon. (A) In the proliferative layer of E12.5 wild-type neocortex, Tbr2 labels cells that are β-III-tubulin negative (red arrowheads). Some of these cells appear to be dividing indicating they are BPCs (red arrowhead containing white m). In the preplate, Tbr2/β-III-tubulin co-expressing postmitotic neurons are observed (yellow arrowhead) as well as β-III-tubulin-expressing neurons which are down regulating or have down regulated Tbr2 expression (white arrowhead). (B) In the proliferative layer of E12.5 *Pax6*^*−/−*^ neocortex, many Tbr2-positive cells are β-III-tubulin-positive (yellow arrowheads) while some are not (red arrowhead). In the preplate, co-expressing (yellow arrowhead) and β-III-tubulin-positive/Tbr2 negative (white arrowhead) cells are seen. (C, D) A 2-h BrdU pulse was used to label cells that are actively cycling in the E12.5 *Pax6*^*+/+*^ and *Pax6*^*−/−*^ telencephalon. (C) In *Pax6*^*+/+*^ telencephalon, Tbr2/BrdU co-labeled BPCs can be observed in the basal ventricular zone and preplate (white arrowheads). (D) In the *Pax6*^*−/−*^ telencephalon, very few of the BrdU-labeled cells express Tbr2 (white arrows). Scale bars: (A, B) = 20 μm; (C, D) = 40 μm.

To determine the nature of the Tbr2-expressing cells in mutant embryos, we first examined expression of a marker of early postmitotic neurons, β-III-tubulin. In the differentiating wild-type preplate, β-III-tubulin-expressing postmitotic neurons were either Tbr2-expressing or Tbr2-non-expressing (Fig. 6A). This is consistent with the previous description of up regulation of Tbr2 in early postmitotic neurons and subsequent downregulation of Tbr2 in these cells as neuronal differentiation proceeds (Englund et al., 2005). Cells of both types were also seen in the *Pax6*^*−/−*^ preplate (Fig. 6B). Beneath the preplate of wild types, many Tbr2-expressing, β-III-tubulin-non-expressing cells were present and, as previously described, had condensed chromatin indicative of cells undergoing mitosis, confirming them as BPCs (Englund et al., 2005) (Fig. 6A). Beneath the preplate in *Pax6*^*−/−*^ cortex, most of the small number of Tbr2-expressing cells were also β-III-tubulin-positive (Fig. 6B), suggesting these cells are mislocated Tbr2-expressing neurons rather than Tbr2-positive BPCs. No Tbr2-positive mitotic figures were observed in mutants.

As Tbr2 is normally expressed in BPCs (Englund et al., 2005), we wanted to determine whether actively cycling Tbr2-expressing cells were present in mutant embryos. A short (2 h) pulse of BrdU was given at E12.5 to label cells during S-phase of the cell cycle (Figs. 6C, D). In wild-type cortex, co-localization of Tbr2 with BrdU was observed frequently in BPCs in all sections through the basal proliferative zone and preplate (Fig. 6C). In contrast, we observed very few BrdU/Tbr2-expressing cells in *Pax6*^*−/−*^ cortex; Fig. 6D shows two examples but most sections contained none. These data indicated either that BPCs do not exist in significant numbers in *Pax6*^*−/−*^ mutants or that cells dividing non-apically do not express Tbr2 in mutants.

**Fig. 7:**
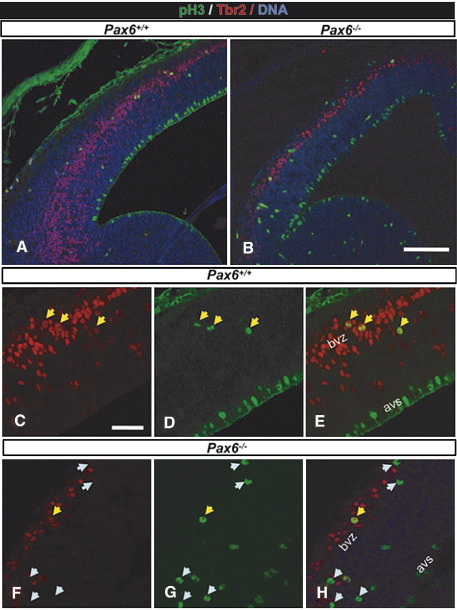
Few abventricular mitoses express Tbr2 in *Pax6*^*−/−*^ mutant telencephalon. Immunohistochemistry for Tbr2 and phosphohistone-3 (pH3) in panels A, C–E *Pax6*^*+/+*^ and (B, F–H) *Pax6*^*−/−*^ E12.5 telencephalon. pH3 labels mitotic cells in late G2/M-phase. (A, C–E) In wild-type telencephalon, cells strongly reactive for pH3 can be observed both at the apical ventricular surface (avs) and within the basal ventricular zone (bvz); all pH3-expressing cells observed in the bvz are also Tbr2-expressing (yellow arrowheads in panels C–E). (B, F–H) In the *Pax6*^*−/−*^ neocortex, there are very few BPCs expressing both Tbr2 and pH3 (yellow arrowhead). In contrast, many pH3-expressing, Tbr2-non-expressing cells (light blue arrowheads) are present within the proliferating zone. Scale bars (A, B) = 100 μm, (C–H) = 80 μm.

Pulse labeling with BrdU does not distinguish clearly between APCs and BPCs, both of which undergo S-phase in a similar location. Therefore, we examined co-expression of the mitotic marker phosphohistone-3 (pH3) and Tbr2 (Fig. 7). pH3 is expressed strongly in cells undergoing late G2/M-phase and is used as a marker of mitosis in cycling cell populations (Scott et al., 2004; Scott et al., 2003). In wild-type neocortex, two pH3-expressing cell populations were observed: (1) pH3-expressing/Tbr2 non-expressing APCs located at the apical ventricular surface; and (2) pH3-expressing/Tbr2-expressing BPCs located in the basal proliferating zone (Figs. 7A, C–E). In wild-type neocortex, all non-apically located pH3-expressing cells were Tbr2 expressing (Figs. 7A, C–E) consistent with the reported expression of Tbr2 in this proliferative population (Englund et al., 2005). In *Pax6*^*−/−*^ cortex (Figs. 7B, F–H), as in wild-type animals, pH3-expressing cells at the apical ventricular surface were always Tbr2-non-expressing. Cells expressing pH3 were also seen at non-apical locations, as in wild-type animals, but the vast majority did not express Tbr2 (Fig. 7G); one double-labeled cell is shown in (Figs. 7F–H), but all other sections showed none. Thus, the early *Pax6*^*−/−*^ cortex contains a population of cells undergoing abventricular mitoses in the location where BPCs are found in wild-type cortex, but hardly any express Tbr2.

### Some cortical cells undergoing abventricular mitoses in *Pax6*^*−/−*^ embryos express the ventral marker Mash1

**Fig. 8:**
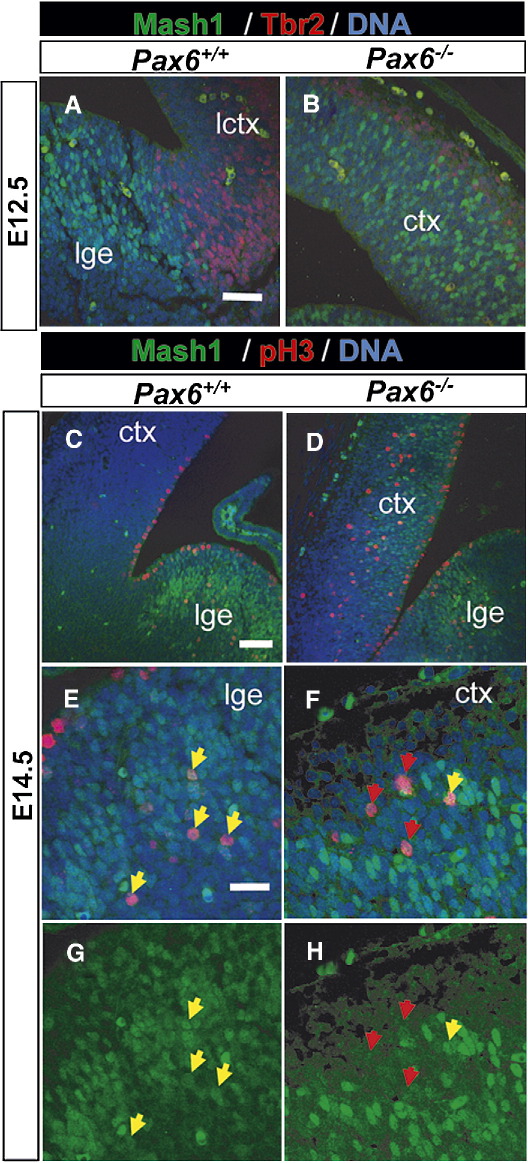
Mash1 is ectopically expressed in some *Pax6*^*−/−*^ neocortical abventricular mitotic cells. (A) At E12.5 in *Pax6*^*+/+*^ telencephalon, Mash1 is restricted to the ganglionic eminences (lge, lateral ganglionic eminence), abutting Tbr2 expression in the developing neocortex (lctx, lateral cortex) at the pallial–subpallial boundary. (B) In E12.5 *Pax6*^*−/−*^ telencephalon, many cells throughout the cerebral cortex (ctx) express Mash1. Tbr2-expressing cells of the mutant preplate do not express Mash1. (C) In E14.5 *Pax6*^*+/+*^ telencephalon, Mash1 expression is restricted to the ganglionic eminences. (D) In E14.5 *Pax6*^*−/−*^ telencephalon, dorsal and ventral telencephalic cells express Mash1. (E,G) Abventricular mitoses in the lateral ganglionic eminence of *Pax6*^*+/+*^ telencephalon express Mash1 (yellow arrows; panel G is the same field as panel E in green channel alone). (F, H) Most pH3-expressing cells undergoing mitosis in abventricular locations in the *Pax6*^*−/−*^ neocortex do not express Mash1 (red arrows); a minority of these cells do express low levels of Mash1 (yellow arrows; panel H is the same field as panel F in the green channel alone). Scale bars (A, B) = 80 μm; (C, D) = 160 μm; (E–H) = 40 μm.

Abventricular mitoses occur with high frequency in normal ventral telencephalon (Sheth and Bhide, 1997), which does not express Tbr2 but does express the transcription factor Mash1 (Fig. 8A) (Guillemot and Joyner, 1993; Lo et al., 1991). Many dividing pH3-positive cells in the wild-type lateral ganglionic eminence express Mash1 (Figs. 8C, E, G). Ectopic dorsal expression of normally ventrally restricted Mash1 mRNA has been reported in *Pax6*^*−/−*^ mutants (Kroll and O'Leary, 2005; Muzio et al., 2002a; Muzio et al., 2002b; Stoykova et al., 2000; Toresson et al., 2000). We confirmed that Mash1 protein is expressed in many cells in the *Pax6*^*−/−*^ cortex (Fig. 8B); this ectopic expression does not co-localize with Tbr2 expression in the preplate. In many sections through *Pax6*^*−/−*^ embryos, we observed examples of abventricular mitoses expressing Mash1 (Figs. 8D, F, H) suggesting at least a partial transformation of the population of abventricularly dividing cells to a ventral telencephalic character.

### Pax6 is required cell autonomously in cortex for expression of Tbr2 and repression of genes normally expressed in the lateral ganglionic eminence

**Fig. 9:**
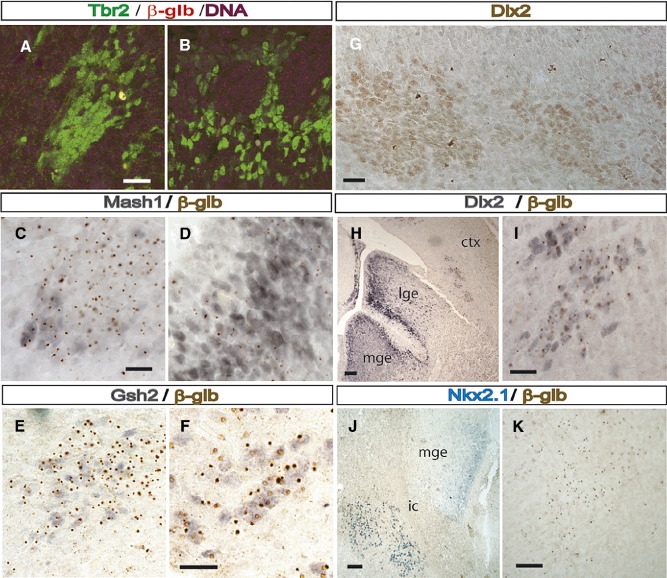
Pax6 is required cell autonomously for Tbr2 expression and repression of lateral ganglionic eminence markers in cortical cells. (A, B) Sections of cortex of *Pax6*^*−/−*^↔*Pax6*^*+/+*^ chimera double-labeled for Tbr2 protein (green) and *Pax6*^*−/−*^ cells (red dots resulting from DNA–DNA in situ hybridization to a reiterated β-globin transgene, β-glb). Most *Pax6*^*−/−*^ cells do not express Tbr2 (Panel A, rostral section; Panel B, central section). (C, D) Sections of cortex (C) and lateral ganglionic eminence (D) of *Pax6*^*−/−*^↔*Pax6*^*+/+*^ chimera double-labeled for Mash1 protein (grey) and *Pax6*^*−/−*^ cells (brown dots). In cortex, Mash1 expression is in *Pax6*^*−/−*^ cells but not in wild-type cells. In the lateral ganglionic eminence, Mash1 is expressed in both wild-type and *Pax6*^*−/−*^ cells. (E, F) Sections of rostral (E) and caudal (F) cortex of *Pax6*^*−/−*^↔*Pax6*^*+/+*^ chimera double-labeled for Gsh2 protein (grey) and *Pax6*^*−/−*^ cells (brown dots). Gsh2 expression coincides with the *Pax6*^*−/−*^ cells. (G) Dlx2-positive cells in the cortex of a *Pax6*^*−/−*^↔*Pax6*^*+/+*^ chimera. (H) Dlx2-positive cells in the lateral ganglionic eminence (lge), medial ganglionic eminence (mge) and cortex (ctx) of a *Pax6*^*−/−*^↔*Pax6*^*+/+*^ chimera; (I) Dlx2-positive cells in the cortex are *Pax6*^*−/−*^ (i.e., Tg-positive). (J) Nkx2.1-positive cells in the medial ganglionic eminence (mge) and around the internal capsule (ic) of a *Pax6*^*−/−*^↔*Pax6*^*+/+*^ chimera; (K) *Pax6*^*−/−*^ (Tg+) cortical cells are Nkx2.1-negative. Scale bars: (A, B, G, K) = 40 μm; (C–F, I) = 20 μm; (H, J) = 60 μm.

In normal mice, Pax6 is downregulated in cells expressing Tbr2 (Englund et al., 2005), raising the question of whether Tbr2 expression in cortical cells requires (i) Pax6 expression in the same cells at an earlier time (i.e., a cell autonomous requirement) or (ii) signals from nearby Pax6-expressing cells (i.e., a cell non-autonomous requirement). To distinguish between these possibilities we examined Tbr2 expression in *Pax6*^*−/−*^ cells that were mixed with wild-type cells in *Pax6*^*−/−*^↔*Pax6*^*+/+*^ chimeras; *Pax6*^*−/−*^ cells were identified by the presence of signal for the β-globin transgene (see Methods; Quinn et al., 1996; Talamillo et al., 2003). We observed many small clusters of *Pax6*^*−/−*^ cells intermingled with and surrounded by wild-type cells expressing Tbr2 (Figs. 9A, B). Almost all *Pax6*^*−/−*^ cells were negative for Tbr2 (Figs. 9A, B) indicating that Pax6 is required cell autonomously by progenitor cells for their normal expression of Tbr2.

**Fig. 10:**
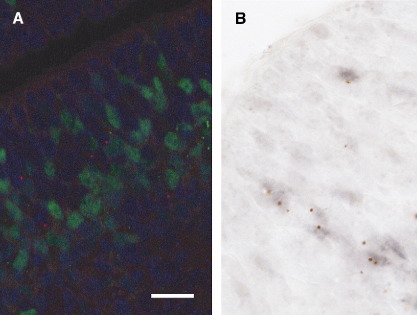
*Pax6*^*−/−*^ cells in the medial regions of the cortex downregulate Tbr2 and upregulate ventral telencephalic markers. (A) Hippocampus of *Pax6*^*−/−*^↔*Pax6*^*+/+*^ chimera double-labeled for Tbr2 protein (green) and *Pax6*^*−/−*^ cells (red dots, as in Fig. 9). (B) Hippocampus of *Pax6*^*−/−*^↔*Pax6*^*+/+*^ chimera double-labeled for Mash1 protein (grey) and *Pax6*^*−/−*^ cells (brown dots). Scale bar = 20 μm.

We then tested whether cells lacking Pax6 in *Pax6*^*−/−*^↔*Pax6*^*+/+*^ chimeras upregulate genes whose expression is normally restricted ventrally in the telencephalon (Fig. 9). We found that most *Pax6*^*−/−*^ cortical cells in chimeras express Mash1 (Fig. 9C), Gsh2 (Figs. 9E, F) and Dlx2 (Figs. 9G–I). Wild-type cortical cells in chimeras do not express these genes (Figs. 9C, E, I), indicating that the presence of mutant cells does not induce expression in wild-type cells. In the ventral telencephalon of chimeras, Mash1, Gsh2 and Dlx2 are expressed by cells of both genotypes (seen for Mash1 in Fig. 9D). Nkx2.1 is also expressed by cells of both genotypes in the proliferative zone and around the internal capsule in the medial ganglionic eminence (Fig. 9J) but not by *Pax6*^*−/−*^ cells in the cortex of chimeras (Fig. 9K). We observed the lack of Tbr2 expression and activation of Mash 1, Gsh2 and Dlx2 (and not Nkx2.1) expression in the *Pax6*^*−/−*^ cells of chimeras throughout the full extent of the cortex, including its most medial regions. Fig. 10 shows examples of the absence of Tbr2 and expression of Mash1 in mutant cells in the hippocampus of chimeras. We conclude that Pax6 has cell autonomous actions within the cortical cells expressing it to activate expression of Tbr2 and to repress expression of genes normally expressed in the lateral ganglionic eminence.

## Discussion

Our aim was to use *in vivo* methods to study early defects in cortical cells lacking Pax6 so as to obtain a clearer picture of the likely primary cell autonomous actions of this transcription factor. Previous studies have demonstrated that, from the onset of corticogenesis, *Pax6*^*−/−*^ cells have a cell autonomous defect in their adhesion properties (Stoykova et al., 1997; Talamillo et al., 2003; Tyas et al., 2003). It is less clear how many of the other cortical defects that have been reported in *Pax6*^*−/−*^ mutants reflect cell autonomous, rather than indirect cell non-autonomous, requirements for Pax6 in the affected processes. Our results indicate that Pax6 is required cell autonomously to regulate the production of correct numbers of dorsal telencephalic cells with the correct identities.

### Pax6 reduces cell cycle exit among early cortical progenitors

Our conclusion that *Pax6*^*−/−*^ cells exit the cell cycle in abnormally large numbers at the onset of corticogenesis rests on evidence from multiple experimental approaches. We showed that, early in corticogenesis, newly divided progenitors do not re-enter S-phase in normal numbers and that there is a corresponding increase in proportions of neurons and reduction in proportions of cortical cells in S-phase. A reduction in size of the pool of mutant progenitors provides an explanation for the underproduction of mutant cells and the reduced size of the mutant cortex. Further evidence supporting this conclusion came from experiments to exclude other possible explanations for underproduction of mutant cells, namely increased cell death and lengthening of the cell cycle.

These findings provide a possible explanation for results described in the study by Heins et al. (2002) which showed that cortical radial glial cells (or APCs) isolated from older, E14, *Pax6*^*−/−*^ embryos have a reduced neurogenic potential. They found that proportions of radial glial progenitors and postmitotic neurons were abnormally low in *Pax6*^*−/−*^ embryos at this later age. This is readily explained by premature exit of progenitors from the cell cycle and the consequent depletion of the neurogenic progenitor pool during the preceding days. In addition, Heins et al. (2002) showed that those radial glial progenitors that are present at E14 produce abnormally small numbers of neuronal clones *in vitro*, suggesting that their neurogenic potential has altered. This might be a direct consequence of the lack of Pax6 in these progenitors. Alternatively, it might be an indirect consequence due, perhaps, to a change in the size of the radial progenitor population altering cell–cell signaling between progenitors and redefining their developmental fates.

Interestingly, an increase in the proportion of reelin-expressing cells has been reported in the *Pax6*^*−/−*^ mutant cortex at E14.5 (Stoykova et al., 2003). Reelin-expressing cells are derived from the earliest postmitotic cells of the neocortex. Again, this finding is compatible with our results: an increased production of postmitotic neurons early in corticogenesis followed by a reduced production later on could explain an increased proportion of reelin-expressing cells by E14.5. Our findings in the cortex have a parallel in the eye, where loss of Pax6 results in precocious neuronal differentiation in the early optic stalk (Philips et al., 2005).

In a previous study we concluded that cell cycle length in the developing E12.5 cortex is shorter than normal in the *Pax6*^*−/−*^ mutant (Estivill-Torrus et al., 2002). An alternative method for analyzing cell cycle length used here indicates that cell cycle lengths are not altered significantly at E10.5 and E12.5 in *Pax6*^*−/−*^ mutants. Importantly, neither study suggests a lengthening of the cell cycle at these ages, an outcome which might have provided an alternative explanation for underproduction of mutant cells. Nevertheless, it is interesting to consider the possible reasons for the discrepancy between the methods. It is most likely that the earlier study overestimated the reduction in cell cycle times in *Pax6*^*−/−*^ mutants. Estivill-Torrus et al. (2002) used the cumulative BrdU incorporation paradigm (Nowakowski et al., 1989), in which BrdU is administered to a cohort of animals for progressively longer periods of time until all progenitors have taken it up. This time is a critical measure in this method (Nowakowski et al., 1989; Takahashi et al., 1995a) and, in common with others who have studied cell cycle times in wild-type cortex, Estivill-Torrus et al. (2002) estimated it by extrapolation using linear regression on data for the proportions of labeled cells after 3–4 BrdU pulses of increasing lengths. Re-analysis of those data using non-linear regression in mutants, which is appropriate if the progenitor cell cycle times are more heterogeneous in mutants (Nowakowski et al., 1989; Takahashi et al., 1995a), gives cell cycle times much closer to wild-type values (as little as 10% different, as opposed to the 40% difference reported by Estivill-Torrus et al., 2002). An interesting inference from this is that cells in the progenitor pool of *Pax6*^*−/−*^ mutant cortex are likely to be more heterogeneous in their cell cycle times, as they are in their abnormal expression of Mash1, which is present in only some mitotic cells.

### Pax6 regulates cell autonomously the identity of dorsal telencephalic progenitors

Pax6 is required for two major features of dorsal telencephalic cell identity, namely the suppression of ventral gene expression and the production of a normal population of Tbr2-expressing BPCs. Previous studies have shown that the cerebral cortex of *Pax6*^*−/−*^ mutants becomes progressively ventralized during the second half of gestation, with increasing proportions of cortical cells expressing markers typical of ventral telencephalic cells (Stoykova et al., 2000; Toresson et al., 2000; Yun et al., 2001; Kroll and O'Leary, 2005). Kroll and O'Leary (2005) showed that this ventralization is due to a change in the fates of cells generated from the cortical proliferative zone. Whether this fate change is induced in cortical cells by defects in interacting cells lacking Pax6 or whether it reflects a cell autonomous requirement for Pax6 for the suppression of ventral fates within cortical cells was not clear. Our data indicate that the defective identities of mutant cortical cells reflect cell autonomous requirements for Pax6 within them. Moreover, the autonomously affected mutant cells were not able to induce expression of ventral markers in surrounding wild-type cells. We conclude that the presence of Pax6 within wild-type cortical cells is necessary to suppress their expression of lateral ganglionic eminence genes. It is not, however, required to prevent their expression of the medial ganglionic eminence marker, Nkx2.1; loss of Pax6 is not sufficient to activate Nkx2.1 expression in dorsal telencephalic cells.

Englund et al. (2005) showed that cortical BPCs express Tbr2 but downregulate Pax6. Cortical BPCs give rise to neurons (Haubensak et al., 2004; Miyata et al., 2004; Noctor et al., 2004) and there is evidence that the superficial layers are generated by these neurons (Tarabykin et al., 2001). Previous studies in *Pax6*^*−/−*^ mutants have shown downregulation of expression of a variety of molecules normally expressed in cortex, including R-cadherin and Svet1 (Stoykova et al., 1997; Tarabykin et al., 2001). In the present study, we identified a loss of Tbr2 in cells deep to the cortical plate, a population that includes cells undergoing abventricular mitoses which would normally express Tbr2 (i.e., BPCs). It is likely that *Pax6*^*−/−*^ BPCs are not correctly specified. Interestingly, whereas Pax6 is required cell autonomously to generate a normal population of Tbr2-expressing BPCs, Caric et al. (1997) showed that E16 *Pax6*^*−/−*^ cortical cells transplanted into a wild-type environment were able to contribute in large numbers to the superficial layers. The function of Tbr2 in cortical development is not clear but it is possible that expression of neither Pax6 nor Tbr2 is required within cortical progenitors for them to generate neurons capable of populating the superficial layers. More likely, these genes may be required to specify the types of cells produced rather than their locations.

The differential expression of transcription factors confers different regional characteristics to progenitor cells in both dorsal and ventral telencephalon (Zaki et al., 2003). Pax6 plays a significant role in regionalization of the developing telencephalon both prior to and during corticogenesis (Bishop et al., 2000; Muzio et al., 2002a; Toresson et al., 2000; Yun et al., 2001). In the developing neocortex, regional differences in cell proliferation may underlie regional differences in the cortical areas produced (Polleux et al., 1997). Tbr2 is expressed at different levels in different parts of the wild-type cortex, exhibiting a matching ^high^lateral to ^low^dorsal gradient of expression to that of Pax6 (present results; Englund et al., 2005). We found that loss of Pax6 expression causes loss of graded Tbr2 expression, the greatest loss occurring in the lateral cortex of the *Pax6*^*−/−*^ mutant telencephalon, where Pax6 is most highly expressed in the wild-type embryo. It seems likely that loss of graded Tbr2 expression contributes to the loss of cortical regionalization observed in the late-gestation *Pax6*^*−/−*^ embryo (Bishop et al., 2000; Muzio et al., 2002a).

## Conclusions

Our findings point to three early cell autonomous roles for Pax6 in developing cortex: (i) maintenance of the size of the cortical progenitor pool; (ii) activation of Tbr2 expression; (iii) repression of genes normally expressed ventrally, in the lateral ganglionic eminence. We suggest that Pax6 is required cell autonomously to keep dorsal telencephalic progenitor cells in the cell cycle and maintain their dorsal identities.

## Figures and Tables

**Table 1:** Average lengths (mean ± SEM) of S-phase (Ts) and total cell cycle (Tc) for *Pax6*^*+/+*^ and *Pax6*^*−/−*^ telencephalon at E10.5 and E12.5 estimated by IdU/BrdU double labeling method

Dorsal telencephalon	Ts (h)	Tc (h)	Ts/Tc
*E10.5*			
*Pax6*^*+/+*^ (*n* = 2)	4.4	7.5	0.58
*Pax6*^*−/−*^ (*n* = 2)	5.0	8.6	0.58

Neocortex	Ts (h) ± SEM	Tc (h) ± SEM	Ts/Tc ± SEM

*E12.5*			
*Pax6*^*+/+*^ (*n* = 3)	5.8 ± 0.78	11.9 ± 1.69	0.48 ± 0.22
*Pax6*^*−/−*^ (*n* = 4)	6.9 ± 0.09	14.4 ± 0.24	0.47 ± 0.61
*p*=	0.15	0.24	0.61

*Hippocampus*			
*Pax6*^*+/+*^ (*n* = 3)	6.9 ± 1.36	17.1 ± 4.18	0.40 ± 0.02
*Pax6*^*−/−*^ (*n* = 4)	7.3 ± 0.45	16.5 ± 1.04	0.44 ± 0.03
*p*=	0.81	0.89	0.47

Data were compared using a Student's *t*-test to obtain *p* values.
